# Enhancing informal workers’ tools to reduce workplace injuries: a quasi-randomized control trial of electronic waste recyclers in Thailand

**DOI:** 10.5271/sjweh.4259

**Published:** 2026-03-01

**Authors:** Abas Shkembi, Emma Linhart, Suzanne Chou, Marianna J Coulentianos, Achyuta Adhvaryu, Jesse Austin-Breneman, Kowit Nambunmee, Richard L Neitzel

**Affiliations:** 1 Department of Environmental Health Sciences, University of Michigan School of Public Health, Ann Arbor, MI, USA.; 2 Department of Mechanical Engineering, University of Michigan College of Engineering, Ann Arbor, MI, USA.; 3 School of Design and Creative Arts, Loughborough University, United Kingdom.; 4 School of Global Policy and Strategy, University of California, San Diego, La Jolla, CA, USA.; 5 Franklin W. Olin College of Engineering, 1000 Olin Way, Needham, MA, USA.; 6 Urban Safety Innovation Research Group (USIR), School of Health Science, Mae Fah Luang University, Chiang Rai, Thailand.

**Keywords:** e-waste, incident, intervention, LMIC, occupational health, safety

## Abstract

**Objectives:**

In low- and middle-income countries (LMIC), there is mixed evidence on the effectiveness of interventions in improving workplace conditions among hazardous industries. In Thailand, a particularly hazardous industry with high injuries is informal electronic waste (e-waste) recycling. We investigated whether developing an optimized tool to dismantle e-waste would reduce injuries.

**Methods:**

We conducted a quasi-randomized control trial to determine the perceptions and efficacy of the optimized tool in reducing worker injuries over three months among 89 workers. The optimized tool for dismantling e-waste was designed following employee and business owner input using conjoint analysis. Workers were quasi-randomized into an intervention (ie, receiving the tool) or control (ie, not receiving) group from an auction. We conducted differences-in-differences Poisson regression to examine differences in self-reported injuries and near misses over three months follow-up between the intervention and control groups.

**Results:**

Among 44 workers who received the tool, workers self-reported that the tool created a safer work environment and reduced near misses, hammer danger, hand vibrations and hand pain. Among 42 workers (21 treatments, 21 controls) with complete information, the intervention reduced self-reported injuries over three months [difference-in-differences: -58%, 95% confidence interval (CI) -19– -79%]. Similar reductions in near misses were observed but not statistically significant (-53%, 95% CI -92–173%).

**Conclusions:**

Our study suggests that meaningful reductions in injury risk for specific types of work can be achieved with co-designed tools optimized to consider inputs from multiple stakeholders. This approach can be especially useful in resource-constrained environments, including working conditions in LMIC.

While the occupational safety of high-income countries such as the United States has improved over the last several decades, occupational injuries and illnesses in many low- and middle-income countries (LMIC) have intensified (1, 2). This disparity is in part driven by the effects of globalization, wherein hazardous jobs have been transferred from high-income countries to LMIC in order for companies to reduce employee wages and workplace regulatory requirements (3). In addition to insufficient safety protections, a lack of occupational regulation enforcement, low awareness of occupational safety risks, and resource constraints, adapting work-related interventions developed in high-income countries to workplaces in LMIC presents further challenges. As such, there has been a lack of interventions designed and implemented to reduce occupational injuries in LMIC.

Informal electronic waste (e-waste) recyclers in Thailand provide an important case study to develop appropriately designed workplace interventions. Discarded products that contain electronic or electrical components (e-waste), is a global threat to human and ecosystem health in low-income countries, which are also disproportionately burdened by the import of e-waste from higher-income countries (4). While e-waste creates much-needed employment opportunities in these countries through the recovery and sale of valuable components from e-waste (eg, metals), such employment is often informal, precarious, and performed in resource-constrained work environments with little-to-no access to personal protective equipment or safety training (5). As a result, e-waste recyclers experience a wide variety of work-related adverse health outcomes, including hearing loss, stress, thyroid dysfunction, and DNA damage (6).

Occupational injuries are also a particular concern. The work environments of informal e-waste recycling varies across different regions of the world, but in Thailand (a middle-income country), workers often dismantle hundreds of pounds of e-waste of different sizes and composition (eg, televisions, computers, washing machines) in their own homes. They typically do not wear or have access to gloves or close-toed shoes to reduce their risk of acute injury. Workers use any available tool (eg, modified lawn mower blades, scissors, hammers and chisels) to dismantle e-waste, and since most recyclers work at their own homes, their work environments are not conducive to proper ergonomic postures (eg, not sitting low to the ground, not being bent over), increasing their risk of chronic injuries such as musculoskeletal disorders. As a result, e-waste workers in many countries experience occupational injuries (7–9). Given the informal, dynamic, and hazardous nature of this important recycling work, interventions to reduce injury burden are needed. The only example of an intervention targeting occupational injuries we identified in a search of the literature – at Agbogbloshie Market in Ghana – was shown to have marginal efficacy (10).

This study sought to address the high prevalence of occupational injuries among informal e-waste recyclers in Thailand by focusing on one point of intervention: the tools used to dismantle e-waste. Using conjoint analysis guided by the preferences of the informal e-waste recyclers themselves, we designed an optimized tool for dismantling e-waste (11). We then conducted a quasi-randomized control trial to determine the perceived performance and efficacy of the optimized tool in reducing worker injuries. We hypothesized that receiving the tool would reduce injuries compared to not receiving the tool.

## Methods

This quasi-randomized control trial (RCT) assessed the number of occupational injuries experienced by informal e-waste recyclers in Thailand before and after an intervention where some recyclers dismantled e-waste using an optimized blade. Injuries were assessed using surveys administered to the participating recyclers. The optimized tool for the quasi-RCT was designed with worker input using conjoint analysis and has been previously detailed (11).

**Figure 1:**
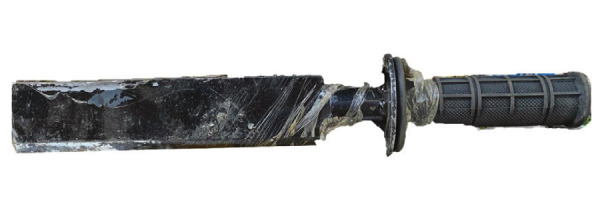
Optimized tool used in the intervention group (handle in side position, 9-inch blade, thick blade, guard).

Briefly, nine e-waste workers provided preliminary input that a blade would be the preferred tool for dismantling e-waste. Subsequently, conjoint analysis was conducted, which estimates the utility for each blade attribute that would enable mechanical engineers to design an optimal tool. The analysis included 98 e-waste workers, who rank-ordered five blade attributes: price, handle position, blade length, blade thickness, and presence of a guard. The optimized tool from the conjoint analysis was a blade design with a 9-inch-long blade of ½ inch thickness on one side, tapered to dull blade on the other side and with a guard and handle (figure 1), which was manufactured locally for under 350 Baht (equivalent to roughly $11.50 USD in 2019). The study was conducted in three stages from August to December 2019: (i) a baseline survey to understand workplace injuries, (ii) an auction to determine the quasi-randomized allocation of the optimized tool to participants, and (iii) a follow-up survey to characterize workplace injuries after tool assignment.

### Study participants

Participants were recruited from a small community in Northeastern Thailand (Kalasin Province) via convenience sampling with assistance from local community health volunteers and in-country researchers from Mae Fah Luang University. This setting was chosen because e-waste recycling has become prevalent in the community as a secondary form of income generation. Eligible participants had to be ≥18 years old, engage in e-waste recycling as a primary or secondary source of income, and primarily dismantle e-waste. Other common activities conducted by e-waste workers, but not assessed here, include collecting, sorting, and burning e-waste. Participants were ineligible if they did not report using a blade or chisel tool to dismantle e-waste. Eligible participants signed an informed consent form prior to enrollment and participation. The University of Michigan approved the research methods in the United States (approval number HUM00114562) and Mae Fah Luang University did so in Thailand (REH-59104).

### Stage 1: Baseline survey

In August 2019, a student from Mae Fah Luang University, accompanied by a local health volunteer and a University of Michigan researcher, administered a baseline survey to each participant. The survey was originally developed in English by University of Michigan researchers with input from Mae Fah Luang University researchers to ensure there was high fidelity when translated into Thai. A Thai- and English-speaking researcher at Mae Fah Luang University translated the English survey, and a Thai and English-speaking researcher at the University of Michigan translated the survey results back into English.

The survey collected information on demographic information, job history, occupational injuries, business management and organization, and income structure. Workplace injuries were assessed by asking “In the last 3 months, about how many total times have you been injured while doing e-waste work?”. Near misses were assessed by asking “In the last 3 months, how often do you think were you almost in accident or almost injured while doing e-waste work?”.

### Stage 2: Auction and quasi-randomization

The auction methodology was built on the Becker-DeGroot-Marschak mechanism (12). The purpose of the auction was to estimate participants’ willingness-to-pay (WTP) for the new tool. Before the auction began, researchers collected a pilot sample of “bids” (ie, how much each individual would be willing to pay for the new tool) from a subset of 20 study participants in order to set the WTP distribution for the tool. At each home during the auction, this distribution was used to randomly select a price draw. This means that the price of the tool varied randomly by household.

In November 2019, researchers visited each participant at their home. Each participant was given US$10 in tickets (each worth US$1) for use in the auction sale. Each auction was held individually, ie, between a single participant and the research staff conducting the auction. The participant was shown the new tool, received an explanation of its potential benefits, and was allowed to experiment and handle the object to assign it some value in their minds. Before the actual auction, we hosted a “practice sale” by providing the participant the opportunity to buy an everyday object with a retail value of US$1. The objective here was only to familiarize the participant with the auction process, as participants had one chance to buy the tool. Participants wrote down on a sheet of paper their maximum WTP for the tool in number of tickets (1–10, in units of US$1). The average WTP was 364 baht (equivalent to US$12 in 2019). This price was compared with the random price chosen by the researchers. If the bid was above the random price, then the person paid the random price (in tickets) to buy the product. If the price was above the participant’s bid, they did not buy the product. Thus, half of participants were randomly assigned to use the optimized tool in in their usual e-waste recycling work for 8–12 weeks until the stage 3 follow-up, while the other half served as controls.

We consider this study to be a quasi-RCT. While participants were allocated into either the intervention (ie, received the tool) or control group, those who had a greater interest in using the optimized tool would have been willing to pay more for it. Thus the method of assigning treatments and controls was not truly random. While a truly random assignment of treatments and controls is ideal for causal inference, in order to balance the interests of our interdisciplinary team in occupational health, economics, and mechanical engineering with the logistical constraints of implementing this study, we coupled the Becker-DeGroot-Marschak mechanism with treatment/control assignment with the intention of accounting for potential treatment bias using inverse probability weighting.

### Stage 3: Follow-up

Accompanied by a local health volunteer and University of Michigan research staff member, a student from Mae Fah Luang University administered a survey in December 2019 to all participants who completed the baseline survey. The survey collected data on use of the new tool (for participants who received the tool), occupational injuries since the beginning of stage 2, income changes, and opinions on tool design. The survey enabled a comparison of outcomes among the intervention group that received the new tool and the group that did not.

### Participant remuneration

Remuneration was in the form of personal protective equipment (PPE) rather than cash in recognition of local Thai customs of gift-giving. Subjects were compensated at the end of each stage for their participation, with total remuneration ranging from PPE worth US$20–30 depending on whether participants received the new tool from the auction.

### Statistical analysis

*Descriptive statistics.* All statistical analyses were conducted in R v.4.4.1. At baseline, we conducted summary statistics (eg, percentages, means) on the sociodemographic characteristics of the participants. We broke down these summary statistics by the intervention and control groups to assess group differences. Among the subset of participants who received the tool, we also assessed their perceptions of the tool’s efficacy on creating a safer workplace and reducing injury risk.

*Difference-in-differences regression modelling.* To test our main hypothesis that recyclers who received the tool would have significantly fewer injuries than those who did not receive it, we conducted two difference-in-differences regression analyses, one each for the outcomes of all injuries and near misses compared at baseline and follow-up. A Poisson regression model was fitted to estimate the relative risk (RR) of injuries. We fit a logistic regression model to estimate the odds ratio (OR) of reporting a near-miss during at least half of a participant’s workdays. Logistic rather than Poisson regression was used as there is likely recall bias for near-miss events; the binary classification of near misses likely reduces the impact of this recall bias on our estimations. The data was stored in panel format for modelling, such that each participant contained two observations: the number of incidents at baseline and endline.

For each model, the main independent variables of interest were the tool intervention assignment and change in time between baseline to endline, as well as an interaction term between the two variables to assess the difference-in-differences. Each model was adjusted for gender to account for potential confounding. Since the tool intervention assignment was quasi-random, we incorporated inverse probability of treatment weighting (IPTW) to ensure exchangeability between those who received the tool and those who did not. The probability of being in the intervention group was estimated using a logistic regression model, with age and household income chosen as predictors after comparing differences in sociodemographic characteristics between the two intervention groups. Inverse probability weights were calculated and were applied to each regression model. The effect estimates are reported according to recommendations by Knol & VanderWeele (13).

## Results

Eighty-nine workers participated in the baseline survey and auction, of which 42 finished the study through follow-up with complete injury information. Among these 42 participants, 21 (50%) received the tool in the auction and 21 did not (50%). The sociodemographic characteristics for the participants with complete information and all enrolled participants are presented in table 1. The intervention group was slightly older [mean 47 (standard deviation (SD) 12) years] than the control group [45 (SD 12) years]. Those in the intervention group had a generally lower household income than those in the control group.

**Table 1:** Descriptive statistical results and comparison of demographics between intervention (received optimized tool) and control (did not receive optimized tool) groups among Thai workers who completed the study or enrolled in the study. [SD=standardized deviation; IQR=interquartile range.]

Characteristic		Age (years)		Household size		Gender (male)		Household income (<10k Baht)		Education (primary or none)		Baseline injury count		Baseline near misses ^a^
Summary statistic		Mean (SD)		Mean (SD)		N (%)		N (%)		N (%)		Median (IQR)		N (%)
Completed study														
	All (N=42)	46 (12)		4.5 (1.9)		22 (52)		14 (33)		19 (47)		0 (1)		19 (45)
	Intervention (N=21)	45 (12)		4.4 (1.7)		8 (38)		6 (28)		8 (38)		1 (1)		11 (52)
	Control (N=21)	47 (12)		4.6 (2.1)		14 (67)		8 (39)		11 (52)		0 (1)		8 (38)
Enrolled in study														
	All (N=89)	48 (10)		4.6 (2.2)		44 (49)		30 (33)		55 (62)		0 (1)		44 (49)
	Intervention (N=36)	46 (12)		4.6 (2.2)		17 (47)		11 (31)		18 (50)		1 (1)		18 (50)
	Control (N=53)	49 (9)		4.6 (2.2)		27 (51)		19 (26)		37 (70)		0 (1)		26 (49)

^a^ As a count of individuals who experienced near missed on more than half of their workdays.

**Figure 2:**
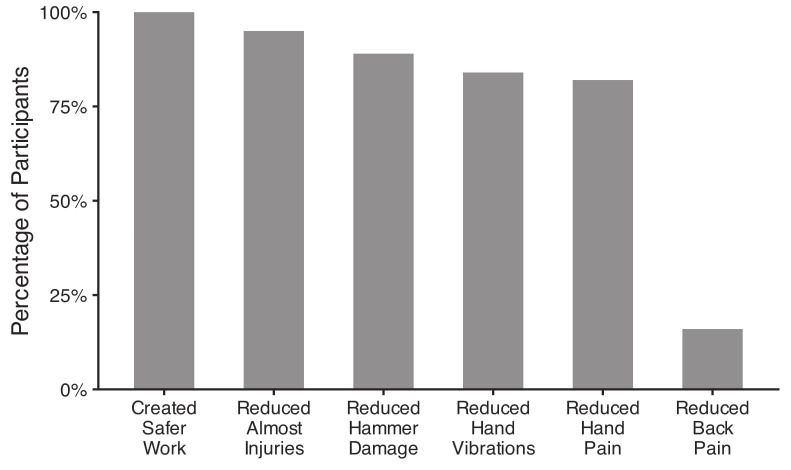
Perceptions of the impact of the tool intervention on workplace hazards among those who received the tool (N=44).

Figure 2 displays the perceptions of the tool intervention on workplace safety among workers who received the tool (N=44) regardless of whether they completed the entire study. All workers (100%) who received the tool reported that it created a safer workplace. The majority of workers reported improvements to injury risk. Around 95% of workers reported a reduction in near misses and 89% reported reduced hammer danger. A similar percentage reported ergonomic improvements from the intervention, including reduced hand vibrations (84%) and reduced hand pain (82%). Only 16% reported that the intervention reduced back pain.

Among the 42 participants with complete information, table 2 presents the risk ratio between the intervention group and the control group over time (baseline to endline) and the risk of all injuries in the previous three months, after controlling for gender and accounting for potential treatment assignment bias (using IPTW). At baseline, workers who received the tool did not have statistically significant differences in all-injuries compared to those who did receive the tool. At follow-up, those who received the tool reported 62% (95% CI 36−78%) fewer injuries in the three months prior compared to baseline (table 2). The effect of the intervention was statistically significant (difference-in-differences: -58%; 95% CI -18−-79%). No statistically significant difference in the odds of near-miss incidents were observed between the two groups (table 3), although a negative relationship was observed as well (difference-in-differences: -53%; 95% CI -92−173%).

**Table 2:** Risk ratios (RR) and 95% confidence intervals (CI) of all injury counts in the last three months by joint effect between study period and intervention status. Model^a^ adjusted for gender. Inverse probability of treatment weights were constructed using a logistic regression model regressing age and income level onto whether participant received a tool to account for potential confounding of who received the tool.

	Baseline		Endline		Study period within strata of intervention status
	RR (95% CI)		RR (95% CI)		RR (95% CI)
Did not receive tool	Reference		0.91 (0.59–1.38)		0.91 (0.59–1.38)
Received tool	0.89 (0.58–1.35)		0.34 (0.19–0.57)		0.38 (0.22–0.64)

^a^ Measure of effect modification on multiplicative scale: RR of interaction term 0.42 (95% CI 0.21–0.82).

**Table 3:** Odds ratios (OR) 95% confidence intervals (CI) of experiencing near-miss incidents on more than half of workdays in the last three months by joint effect between study period and intervention status. Model^a^ adjusted for gender. Inverse probability of treatment weights were constructed using a logistic regression model regressing age and income level onto whether participant received a tool to account for potential confounding of who received the tool.

	Baseline		Endline		Study period within strata of intervention status
	OR (95% CI)		OR (95% CI)		OR (95% CI)
Did not receive tool	Reference		0.65 (0.18–2.30)		0.65 (0.18–2.30)
Received tool	1.65 (0.48–5.83)		0.50 (0.14–1.77)		0.30 (0.09–1.01)

^a^ Measure of effect modification on multiplicative scale: odds ratio of interaction term 0.47 (95% CI0.08–2.73).

## Discussion

This study employed a quasi-RCT to evaluate the efficacy of a tool intervention designed to reduce occupational injuries among informal e-waste dismantlers in Northeastern Thailand. Participants either received the intervention or served as controls. Workplace injuries were assessed at baseline and approximately three months post-intervention. As the intervention group showed a statistically significant reduction in injuries over time, the results support our hypothesis that receiving the tool reduced occupational injuries compared to not receiving it. While there were no statistically significant differences detected between the groups in terms of near-miss incidents, an overall reduction was nevertheless observed. These findings suggest that our engagement with informal e-waste recyclers to develop and implement an optimized tool design was effective in reducing workplace injuries.

At the time of this study, there appears to have been only one other interventional study designed to reduce work-related injuries among informal e-waste workers in LMIC. That intervention – the introduction of a mechanized wire-stripping machine for informal e-waste recycling in Agbogloshie Market in Accra, Ghana – was intended to reduce injuries as well as exposure to air contaminants associated with burning (rather than stripping) e-waste cables. The machine was considered a failure due to lack of take-up from the e-waste recycling workforce, which was in turn partly due to a lack of reliable electrical power to run the machine (10). Several other workplace interventional studies in Thailand (not among e-waste recyclers) yielded similar findings of reduced workplace injuries and improved workplace conditions (ie, heat and lighting) (14, 15). However, there is mixed evidence on the general effectiveness of workplace interventions in LMIC that seek to improve other health endpoints and working conditions. For example, several studies have reported that workplace conditions and health behaviors (eg, accidents, tobacco use, HIV prevention) have deteriorated or worsened after implementation of workplace health promotion interventions (16–19).

A 2020 systematic review on workplace health promotion in LMIC identified seven key factors influencing the effectiveness of interventions: (i) long intervention time; (ii) meeting worker needs; (iii) active education strategies; (iv) commitment from workplace leaders; (v) involvement of workers; (vi) outside help to sustain intervention; and (vii) difficulty of business operations (20). Our study considered a few of these factors (ie, factors 2, 4, 5, and 7) in the intervention design, which may explain why we observed reduced workplace injuries. The overall success of the intervention is likely attributable at least in part to the previous studies we conducted prior to this interventional study with this community of e-waste workers and the conjoint analysis that gathered workers’ preferences in the design of the intervention tool (11, 21–25). While previous studies have demonstrated that a sustained, long-term intervention is an important factor influencing effectiveness (20), the acute nature of our study’s outcome (ie, injuries) made the relatively short intervention time of three months appropriate. On the other hand, due to limited funding, our study lacked active education strategies, commitment from workplace leaders, and outside help to sustain the intervention beyond a few months. Thus, while our intervention may have reduced injuries in the short term, it is unclear that a long-term reduction in injuries would persist, particularly in the event that the tool breaks and is no longer usable (which happened to a handful of participants over the short duration of the study).

### Limitations

The relatively small sample size of participants who completed both baseline and endline surveys with all necessary information (N=42) limits the statistical power of our findings. Due to the nature of the auctioning process, participants with a greater interest in the tool may have been more likely to receive the tool, leading to potential self-selection into the intervention group, causing potential bias. While we employed IPTW to minimize this bias and ensure exchangeability between the two groups in this intervention study, there may be other confounding factors that our study was not able to measure. Due to the informal and fluid nature of e-waste recycling and study recruitment, approximately half of initial participants who enrolled at baseline were not included in the analyses due to lack of follow-up. This potentially introduced selection bias, although baseline participant characteristics measured in this study (age, gender, household income, education, injuries) did not vary substantially between all participants and the subset of participants with complete data.

The outcomes of this study relied on self reporting of injuries and near misses. Injuries can be traumatic and easier to recall, likely resulting in less measurement error which can explain why we were able to observe statistically significant differences between those who received and did not receive the tool. However, those who received the tool could have over-reported how well the tool worked because it can be difficult to get critical feedback from users in a design process, notably in cross-cultural settings with power dynamics at play. This phenomenon was potentially limited by the fact that the follow-up survey was conducted by different people to those who brought the tools. Near misses, as compared to injuries, are inherently less memorable than injury events and may therefore be more difficult to recall after three months. We attempted to reduce potential measurement error by categorizing workers based on whether they reported high or low near misses (ie, above or below more than half of workdays). Future studies may consider using daily diaries at the end of each workday to obtain more reliable near miss data. Regardless, the total sample size with complete information on near misses was small (N=22), and the combination of high measurement error due to recall bias and low sample size may in part explain the wide confidence intervals in the near miss regression models.

Our study suggests that low-cost occupational safety interventions, co-designed with the workers they are intended to protect, can result in meaningful reductions in injury risk (at least in the short-term) among workers in resource-constrained settings. Additionally, the experience of the study team evaluating informal e-waste recycling work on three continents (Africa, Asia, and South America) indicates that the dismantling activities associated with e-waste recycling have similarities across the three disparate locations (8, 21, 26). Rather than developing multiple interventions to address injury risk reduction in this vulnerable workforce, interventions like the one evaluated here may be scalable to different locations with no or minimal modifications. While the accuracy of this notion must be assessed with additional research, if it is shown to be correct this could further help reduce the costs associated with interventions to reduce the burden of occupational injuries in informal e-waste workers. Even with such scalability, though, innovations are needed to ensure that this and other interventions are sustainable over the long term. One factor that will likely be important to ensuring the sustainability of such efforts is the local availability of workshops that can fabricate safe tools; our tool was designed with this goal in mind.

### Concluding remarks

Informal e-waste workers around the world face injury risks every day and subsequently bear a high burden of occupational injuries. The intervention described here demonstrates that a thoughtfully designed intervention (here, in the form of a purpose-built tool co-designed with input from workers and occupational safety researchers) can significantly reduce occupational injury risk. While this tool was intended to reduce injuries experienced during only one of the many activities associated with e-waste recycling (ie, dismantling), this activity is one of the highest-risk activities associated with recycling electronics, and as such may play a disproportionate role in the injury experience of these workers. Additional interventional innovations are needed to further reduce the risk of injuries and illnesses among this critical but understudied and underserved sustainability workforce.

## Data Availability

Data can be provided by the corresponding author upon reasonable request.

## References

[1] Hogstedt C, Wegman DH, Kjellstrom T. The Consequences of Economic Globalization on Working Conditions, Labor Relations, and Worker’s Health. In: Kawachi I, Wamala S, editors. Global Health. Oxford: Oxford University Press; 2007. p. 138–57.

[2] World Health Organization. Global strategy on occupational health for all: The way to health at work. Recommendations of the Second Meeting of the WHO Collaborating Centres in Occupational Health, October 11–14, Beijing, China. 1995.

[3] Sorensen G, Nagler EM, Pawar P, Gupta PC, Pednekar MS, Wagner GR. Lost in translation: the challenge of adapting integrated approaches for worker health and safety for low- and middle-income countries. PLoS One 2017 Aug;12(8):e0182607. 10.1371/journal.pone.0182607 28837688 PMC5570315

[4] Robinson BH. E-waste: an assessment of global production and environmental impacts. Sci Total Environ 2009 Dec;408(2):183–91. 10.1016/j.scitotenv.2009.09.044 19846207

[5] Munni MN, Karim MR, Haque M, Khan S, Khan MA, Hossain I. Awareness, Safety Practices and Associated Factors Among E-Waste Recycling Workers in Bangladesh. Environ Health Insights 2024 Aug;18:11786302241271555. 10.1177/11786302241271555 39148587 PMC11325305

[6] Okeme JO, Arrandale VH. Electronic Waste Recycling: Occupational Exposures and Work-Related Health Effects. Curr Environ Health Rep 2019 Dec;6(4):256–68. 10.1007/s40572-019-00255-3 31734812

[7] Fischer D, Seidu F, Yang J, Felten MK, Garus C, Kraus T et al. Health Consequences for E-Waste Workers and Bystanders-A Comparative Cross-Sectional Study. Int J Environ Res Public Health 2020 Feb;17(5):1534. 10.3390/ijerph17051534 32120921 PMC7084368

[8] Burns KN, Sayler SK, Neitzel RL. Stress, health, noise exposures, and injuries among electronic waste recycling workers in Ghana. J Occup Med Toxicol 2019 Jan;14(1):1–11. 10.1186/s12995-018-0222-9 30647766 PMC6327403

[9] Ohajinwa CM, van Bodegom PM, Vijver MG, Olumide AO, Osibanjo O, Peijnenburg WJ. Prevalence and injury patterns among electronic waste workers in the informal sector in Nigeria. Injury Prevention [Internet]. 2017;injuryprev--2016--042265. Available from: http://injuryprevention.bmj.com/lookup/doi/10.1136/injuryprev-2016-042265 10.1136/injuryprev-2016-042265 28679520

[10] Schneider AF. Managing change in operations: The case of the wire stripping machine in Agbogbloshie, Ghana. 26th Conference of the European Operations Management Association. Helsinki, Finland; 2019. p. 1479–88.

[11] Coulentianos MJ, Arezoomand M, Chou S, Austin-Breneman J, Adhvaryu A, Nambunmee K et al. Product representations in conjoint analysis in an LMIC setting: comparing attribute valuation when three-dimensional physical prototypes are shown versus two-dimensional renderings. Dev Eng 2021;6:100063. 10.1016/j.deveng.2021.100063

[12] Becker GM, DeGroot MH, Marschak J. Measuring utility by a single-response sequential method. Behav Sci 1964 Jul;9(3):226–32. 10.1002/bs.3830090304 5888778

[13] Knol MJ, VanderWeele TJ. Recommendations for presenting analyses of effect modification and interaction. Int J Epidemiol 2012 Apr;41(2):514–20. 10.1093/ije/dyr218 22253321 PMC3324457

[14] Krungkraiwong S, Itani T, Amornratanapaichit R. Promotion of a healthy work life at small enterprises in Thailand by participatory methods. Ind Health 2006 Jan;44(1):108–11. 10.2486/indhealth.44.108 16610544

[15] Manothum A, Rukijkanpanich J. A participatory approach to health promotion for informal sector workers in Thailand. J Inj Violence Res 2010 Jun;2(2):111–20. 10.5249/jivr.v2i2.36 21483207 PMC3134908

[16] Anthony D, Dyson PA, Lv J, Thankappan KR, Fernández MT, Matthews DR. Reducing Health Risk Factors in Workplaces of Low and Middle-Income Countries. Public Health Nurs 2015;32(5):478–87. 10.1111/phn.12189 25801204

[17] Nguyen TP, Khai TT. An evaluation of the Participatory Action-Oriented Training (PAOT) program in small enterprises in Vietnam. J Occup Health 2014;56(4):309–16. 10.1539/joh.13-0063-FS 24872195

[18] Richter K, Phillips SC, McInnis AM, Rice DA. Effectiveness of a multi-country workplace intervention in sub-Saharan Africa. AIDS Care 2012;24(2):180–5. 10.1080/09540121.2011.596513 21777086

[19] Liau SY, Hassali MA, Shafie AA, Ibrahim MI. Assessing quality of a worksite health promotion programme from participants’ views: findings from a qualitative study in Malaysia. Health Expect 2014 Feb;17(1):116–28. 10.1111/j.1369-7625.2011.00742.x 22050457 PMC5060703

[20] Pham CT, Phung D, Nguyen TV, Chu C. The effectiveness of workplace health promotion in low- and middle-income countries. Health Promot Int 2020 Oct;35(5):1220–9. 10.1093/heapro/daz091 31495871

[21] Seith R, Arain AL, Nambunmee K, Adar SD, Neitzel RL. Self-reported health and metal body burden in an electronic waste recycling community in Northeastern Thailand. J Occup Environ Med 2019 Nov;61(11):905–9. 10.1097/JOM.0000000000001697 31464817 PMC12206442

[22] Shkembi A, Nambunmee K, Jindaphong S, Parra-Giordano D, Yohannessen K, Ruiz-Rudolph P et al. Work Task Association with Lead Urine and Blood Concentrations in Informal Electronic Waste Recyclers in Thailand and Chile. Int J Environ Res Public Health 2021 Oct;18(20):10580. 10.3390/ijerph182010580 34682326 PMC8535566

[23] Neitzel RL, Sayler SK, Arain AL, Nambunmee K. Metal levels, genetic instability, and renal markers in electronic waste workers in Thailand. Int J Occup Environ Med 2020 Apr;11(2):72–84. 10.34172/ijoem.2020.1826 32218555 PMC7205511

[24] Arain AL, Neitzel RL, Nambunmee K, Hischier R, Jindaphong S, Austin-Breneman J et al. Material flow, economic and environmental life cycle performances of informal electronic waste recycling in a Thai community. Resour Conserv Recycling 2022 May;180:106129. 10.1016/j.resconrec.2021.106129

[25] Chou S, Arezoomand M, Coulentianos MJ, Nambunmee K, Neitzel R, Adhvaryu A et al. The Stakeholder Agreement Metric: Quantifying Preference Agreement Between Product Stakeholders. J Mech Des 2021 Mar;143(3): 10.1115/1.4049315

[26] Yohannessen K, Pinto-Galleguillos D, Parra-Giordano D, Agost A, Valdés M, Smith LM et al. Health Assessment of Electronic Waste Workers in Chile: participant Characterization [Internet]. Int J Environ Res Public Health 2019 Jan;16(3):386. 10.3390/ijerph16030386 30700055 PMC6388190

